# Unexpected Botulinum Toxin Effect on Neck Rejuvenation After a Jawline Lifting Procedure: A Case Report

**DOI:** 10.7759/cureus.115940

**Published:** 2026-09-08

**Authors:** Monica Espinosa

**Affiliations:** 1 Aesthetic Medicine, Universidad Andrés Bello, Santiago, CHL

**Keywords:** botulinum toxin, facial skin, jawline, neck rejuvenation, platysma muscle

## Abstract

Aging of the platysma muscle significantly contributes to the loss of mandibular contour definition and the vertical platysmal bands aspect, negatively affecting the neck appearance. Botulinum toxin has been widely used for facial rejuvenation, including jawline lifting.

We described the case of a 51-year-old woman who underwent lower-face neuromodulator treatment with prabotulinumtoxinA (Nabota®, Daewoong, Seoul, South Korea). Nine superficial injection points per hemiface were applied along the mandibular and submandibular regions. Three-dimensional stereophotogrammetry (LifeViz® Mini, QuantifiCare, Biot, France) was performed before treatment and 15 days post-procedure to quantify the soft tissue displacement and volumetric changes.

Clinical evaluation demonstrated a visible reduction of platysmal bands during muscle activation and improved mandibular contour definition. The 3D analysis revealed a volume reduction of 2.4 mL in the right upper neck region and 6.32 mL along the lateral cervical area. No adverse events were reported.

These findings suggest a distal functional effect on the platysma muscle, highlighting a potentially safer and lower-dose approach for neck rejuvenation. Further studies are required to validate these results.

## Introduction

The anterior neck region is covered by the superficial platysma muscle, whose aging has a visible impact on neck appearance [1]. During aging, the muscle becomes thinner, and its continuous contraction affects the cervicomental angle and reduces mandibular contour definition [2]. The loss of collagen and elastin in the skin, together with its direct contact with the contracted platysma muscle, makes the muscular appearance more visible as vertical bands (called platysmal bands), exacerbating skin laxity and wrinkles [3].

Botulinum toxin is used for facial rejuvenation by temporarily relaxing selected facial muscles from the upper to the lower third of the face [4]. Jawline (Nefertiti) lifting was first described by Dr. Philip Levy in 2007 [5]. This technique considers the application of botulinum toxin along and under each mandible and to the upper part of the posterior platysmal band [5]. The aesthetic outcome is primarily assessed by improvement of the jawline definition and reduction of the cervicomental angle. When neck rejuvenation is the primary objective, the platysma muscle is the principal and direct target of botulinum toxin treatment [6,7]. However, no studies have quantitatively evaluated the effect of a Nefertiti lift performed without direct neck injection on the platysmal bands appearance and neck contour.

In the present clinical case, a beneficial effect of a mandibular lift with botulinum toxin on both platysmal band prominence and neck contour was observed, despite the absence of direct botulinum toxin injection into the neck.

## Case presentation

A 51-year-old woman with no medical history of chronic diseases, no tobacco use, and no reported allergies expressed her intention to undergo facial rejuvenation by tissue repositioning. The patient has prior experience with aesthetic medical treatments, including botulinum toxin injections in the upper third of the face and hyaluronic acid fillers.

Before treatment, the patient presented with mild-to-moderate cervical skin laxity, characterized by a subtle loss of skin firmness and soft tissue support in the submental and upper cervical region, without marked redundant skin folds. The cervicomental angle was mildly blunted, resulting in a less defined transition between the submental region and the anterior neck. Dynamic assessment demonstrated moderate platysmal banding (Grade 2 according to the Validated Photonumeric Platysmal Bands Scale [8]). In addition, the mandibular contour showed moderate loss of definition, particularly along the prejowl and lateral jawline (Grade 2 according to the Merz Jawline Assessment Scale [9]). The patient’s primary concern was the progressive loss of jawline definition and the increasing visibility of platysmal bands, contributing to a less defined cervicomental contour and a less youthful appearance of the lower face. The patient provided written consent for the procedure and for the use of clinical images for academic and scientific purposes.

Prior to the aesthetic treatment, a 70% alcohol solution was applied using unidirectional movements from the central region of the face toward the periphery to reduce the superficial microbial load and minimize the risk of infectious complications. Complete evaporation of the solution was allowed before performing the anatomical marking of the injection points.

Regarding the superficial application of the neuromodulator, nine injection points (in green) were defined per hemiface (Figure 1). Considering the lower third of the face, the first point was established at 2 mm from the medial vertical line to modulate the mentalis muscle, and the second point was established at a caudal and vertical location relative to the oral commissure to modulate the depressor anguli oris muscle. Subsequently, three additional points were determined, distributed equidistantly in a posterior direction toward the mandibular angle [5]. This scheme was then replicated at a second submandibular level, placing four additional points approximately 2 cm caudal to the mandibular border (Figure 1) [10,11]. The black dot in Figure 1 represents the point of botulinum toxin injection in the upper third of the face that completes the rejuvenation treatment in the whole face.

**Figure 1:**
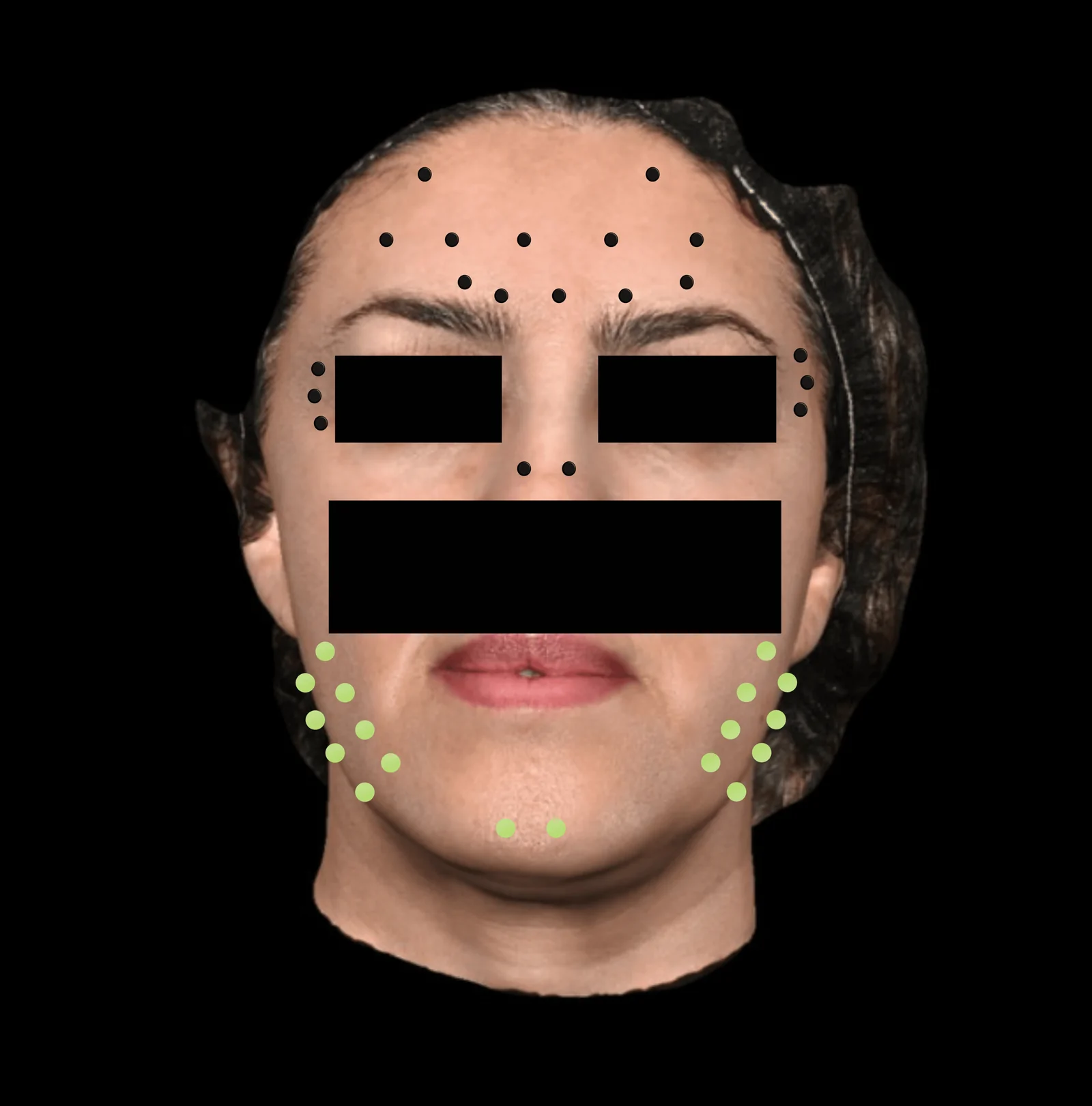
Description of injection points of botulinum toxin. Green points show the location of injection for the jawline lifting treatment.

At each point (green dot), we infiltrated 2.5 IU of prabotulinumtoxinA (Nabota®, Daewoong, Seoul, South Korea) in a volume of 0.07 mL, reconstituted at a ratio of 2.5 mL per 100 IU. The administration was performed using a syringe with a 31G needle directed toward the auricular lobule, maintaining a strictly superficial plane to reach the supramandibular segment of the platysma muscle.

Figure 2 illustrates the patient activating the platysma muscle before and after botulinum toxin treatment. Muscle relaxation results in a visible reduction of platysmal bands in the neck. Platysmal bands are identified by asking the patient to voluntarily contract the platysma muscle.

**Figure 2:**
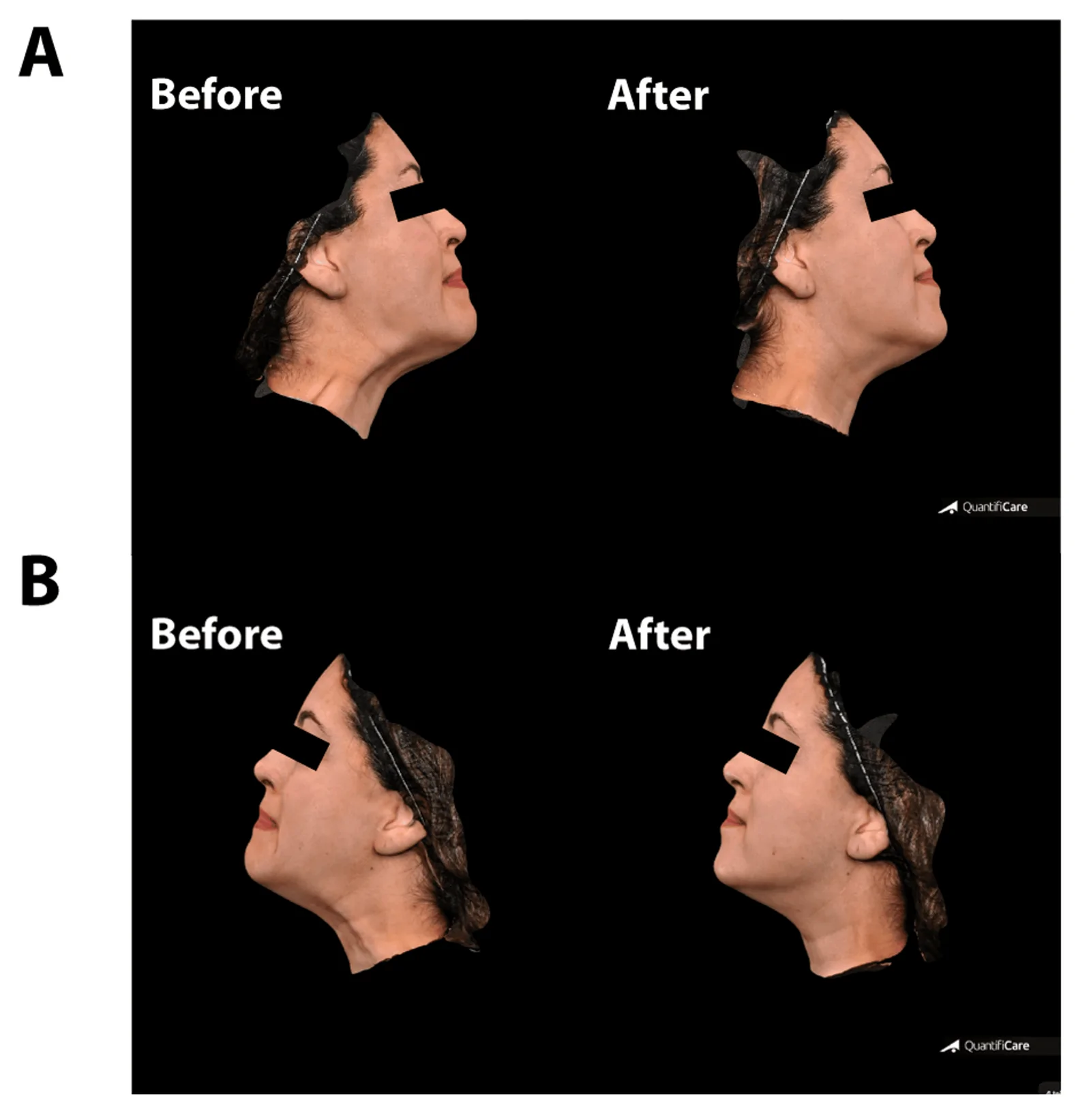
Platysmal bands appearance after muscle activation before and after aesthetic treatment on both sides of the face: right (A) and left (B).

To quantify the effect of the aesthetic procedure on the platysma muscle, images were acquired using a three-dimensional facial scanning system of passive stereophotogrammetry (3D LifeViz® Mini, QuantifiCare, Biot, France) before treatment and 15 days post-procedure. Clinical photographs were obtained using a standardized protocol to minimize variations in patient positioning, lighting, and acquisition geometry. All images were captured in the same room, with the doors closed and under a single artificial light source. Patients were positioned at the same location, using the wall and floor as reference points, without body support. Head position and gaze direction were standardized by asking patients to fix their gaze on a black circle placed at a constant distance. Camera positioning was standardized using the software guidance system, with camera-to-patient distance verified by alignment indicators and horizontal leveling guides. Manufacturer-recommended acquisition settings were used. Four images were obtained: frontal, right oblique, left oblique, and inferior views. Two trained operators followed the same positioning and acquisition protocol. Patients were instructed to remain still with their gaze fixed, and a three-second countdown was performed before each capture. Flash was used for all images to ensure consistent illumination.

The obtained stereoscopic images were automatically integrated using DermaPix database software (QuantifiCare, Biot, France), generating a 3D reconstruction. A comparative analysis was conducted using LifeViz®, quantifying soft-tissue displacement and volumetric variations. The results were displayed using a colorimetric scale, with volume gain or loss (in milliliters) represented in yellow or green, respectively. The patients did not experience any adverse events from the procedure. This method was previously used in skin rejuvenation treatments [12].

In Figure 3, we showed the LifeViz® results quantifying volume differences in the neck area following botulinum toxin treatment. To facilitate identification of platysmal bands, the patient is instructed to voluntarily contract the platysma muscle. A volume reduction of 2.4 mL was measured in the upper region on the right side (A), while a decrease of 6.32 mL was observed along the neck in the lateral view (B).

**Figure 3:**
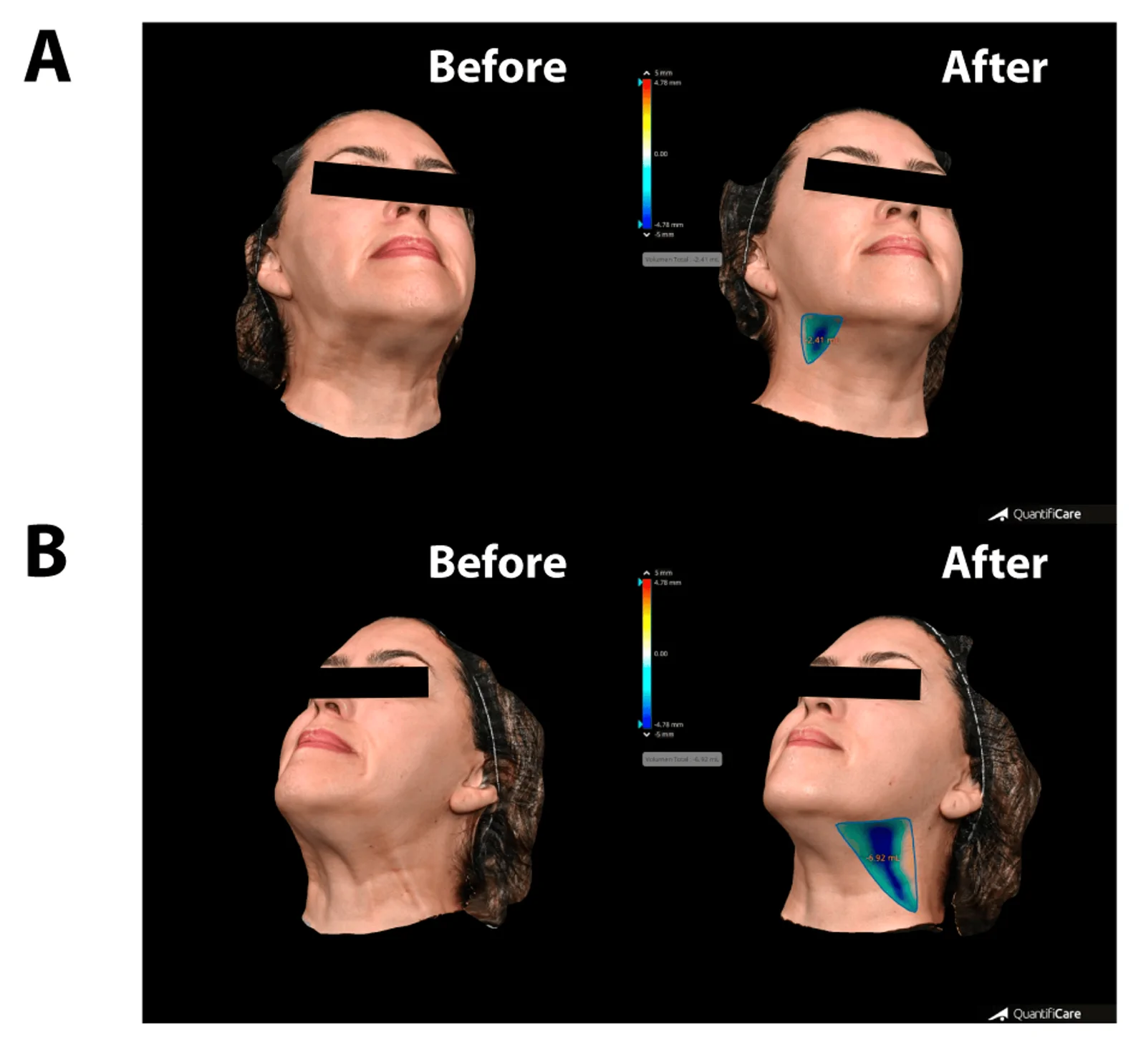
Neck contour appearance quantification after platysma muscle activation on both sides of the neck: right (A) and left (B).

The patient reported a perceived reduction in volume in the mandibular and jowl regions, together with improved jawline definition. She perceived her facial appearance as more refined while maintaining a natural-looking outcome and reported a high level of satisfaction with the treatment results.

## Discussion

Our facial soft tissue repositioning treatment using botulinum toxin induced a direct rejuvenating effect on the mandibular contour and an indirect effect on the neck. This may be explained by mechanisms extending beyond the primary injection site. Ramirez-Castañeda et al. reviewed evidence highlighting that botulinum toxin can produce functional effects in adjacent and even distant muscles through processes of diffusion, spread, and migration, suggesting that its biological activity is not always confined to the targeted muscle [13]. Moreover, Kaya et al. indicated that the effects of botulinum toxin may involve alterations in intermuscular mechanisms, that is, modifying the force balance between functionally antagonistic muscles [14]. Given the anatomical and functional continuity between the mandibular depressor muscles and the platysma, relaxation of the lower facial musculature may indirectly reduce platysmal activity and tension. Although the precise pathway remains uncertain, these findings support the hypothesis that botulinum toxin may exert clinically relevant effects on anatomically and functionally related muscle groups, even in the absence of direct treatment. 

Since direct infiltration of the platysmal bands was unnecessary, clinically effective results were achieved with fewer injection points and a reduced total dose. In addition, this approach reduces the risk of adverse events such as hematoma formation and decreases patient discomfort associated with botulinum toxin injection in the neck [5]. 

The use of three-dimensional stereophotogrammetry (LifeViz® Mini) allowed objective quantification of cervical contour changes following treatment, overcoming some of the limitations associated with conventional photographic assessment. In addition, colorimetric surface mapping facilitated visualization of subtle topographic modifications that may not be detectable through standard clinical examination.

The distal effect of botulinum toxin on the platysma muscle had not been previously quantified, opening a new area of research for optimizing the use of botulinum toxin in aesthetic treatment of the neck.

## Conclusions

As a preliminary result, botulinum toxin treatment for mandibular lifting may induce beneficial effects beyond the primary injection site, contributing to improvement in neck contour and reduction of platysmal band appearance. Further studies with larger sample sizes are required to confirm these observations and clarify the underlying mechanisms.
